# A High-Content Larval Zebrafish Brain Imaging Method for Small Molecule Drug Discovery

**DOI:** 10.1371/journal.pone.0164645

**Published:** 2016-10-12

**Authors:** Harrison Liu, Steven Chen, Kevin Huang, Jeffrey Kim, Han Mo, Raffael Iovine, Julie Gendre, Pauline Pascal, Qiang Li, Yaping Sun, Zhiqiang Dong, Michelle Arkin, Su Guo, Bo Huang

**Affiliations:** 1 Joint Graduate Program in Bioengineering, University of California, San Francisco and University of California, Berkeley, California, United States of America; 2 Small Molecule Discovery Center, University of California, San Francisco, San Francisco, California, United States of America; 3 Department of Pharmaceutical Chemistry, University of California, San Francisco, San Francisco, California, United States of America; 4 Department of Bioengineering and Therapeutic Sciences, University of California, San Francisco, San Francisco, California, United States of America; 5 Department of Biochemistry and Biophysics, University of California, San Francisco, San Francisco, California, United States of America; National Institutes of Health, UNITED STATES

## Abstract

Drug discovery in whole-organisms such as zebrafish is a promising approach for identifying biologically-relevant lead compounds. However, high content imaging of zebrafish at cellular resolution is challenging due to the difficulty in orienting larvae *en masse* such that the cell type of interest is in clear view. We report the development of the multi-pose imaging method, which uses 96-well round bottom plates combined with a standard liquid handler to repose the larvae within each well multiple times, such that an image in a specific orientation can be acquired. We have validated this method in a chemo-genetic zebrafish model of dopaminergic neuron degeneration. For this purpose, we have developed an analysis pipeline that identifies the larval brain in each image and then quantifies neuronal health in CellProfiler. Our method achieves a SSMD* score of 6.96 (robust Z’-factor of 0.56) and is suitable for screening libraries up to 10^5^ compounds in size.

## Introduction

The drug discovery process typically involves screening large libraries of compounds using *in vitro* or cell culture-based disease models. Using these simplified models allows for expedited assessment of compound binding and efficacy, but makes other drug parameters, such as *in vivo* efficacy and toxicity, absorption, distribution, metabolism, and excretion (ADME), difficult to assay [1]. Thus, this approach often results in final stage compounds that fail to show efficacy in whole-organism disease models or have unwanted toxicity [2]. In contrast, high-throughput screening at the whole-organism level can assay both compound effectiveness and ADME; moreover, it can be performed in the absence of a known target [3]. Zebrafish (*Danio rerio*) are particularly well suited for high-throughput screens. As a vertebrate genetic model organism, zebrafish are simple to breed and raise, with optically transparent embryos and larvae that are compatible with liquid handling technologies [4–6] [7,8]. However, the dearth of high-throughput imaging techniques for zebrafish is a major roadblock in the broad utilization of zebrafish models in drug discovery.

A number of small molecule-based screens in zebrafish have been reported [9–11]. The majority of screens image the fish at relatively low resolution, for example, quantifying bulk fluorescence using plate readers [12] or movement (behavior) using low resolution cameras [13–15]. However, high content imaging with cellular level resolution is required for many applications. For instance, quantification of neuronal loss or regeneration requires imaging a specific anatomical region at single-neuron resolution. This type of high resolution imaging is challenging due to the fact that the larvae are complex three-dimensional objects in which the “pose” of the larvae is critical. In the case of imaging neuronal loss, the larval brain can be imaged only when the dorsal side of the larvae is oriented towards the microscope objective. Otherwise, the heavily pigmented eyes obscure clear views of the brain. In our previous screen [16], larvae were manually positioned and imaged on a fluorescent microscope, which was labor intensive and slow (~20–50 compounds a day). One solution to the posing problem is the Vertebrate Automated Screening Technology (VAST) system, a dedicated instrument which uses a capillary tube and stepper motors with imaging feedback to precisely rotate a larva into a specified position [17,18]. However, such solutions can be expensive and space consuming.

We are particularly interested in using zebrafish as a model system for the study of neurodegenerative diseases. Previously, we have developed a chemo-genetic zebrafish model of dopaminergic (DA) neuron degeneration, which occurs in Parkinson’s disease. In this model, we selectively express nitroreductase (NTR) and a mCherry marker in DA neurons. When zebrafish larvae are treated with the harmless prodrug metronidazole (Mtz), the NTR within the neuron converts Mtz into a cytotoxin, causing cell type-specific ablation [19,20]. This model system allows us to screen for compounds that protect DA neurons from degeneration or stimulates their regeneration. To enable such screening, a high-content imaging solution with cellular resolution is required to quantify the health of DA neurons in treated fish larvae.

**Fig 1:**
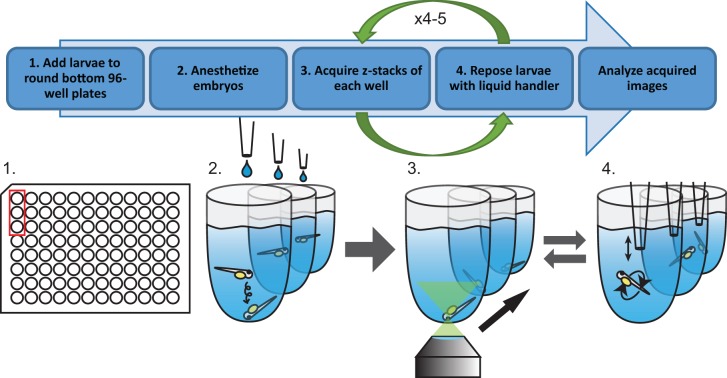
Overview of the multi-pose imaging method.

Here, we report a solution for high-resolution, high-content zebrafish larva imaging that requires no additional equipment other than what is already present in a standard small molecule screening facility. Our solution, the multi-pose imaging method, employs a liquid handler to reposition zebrafish larvae in a 96 well plate, and is compatible with ordinary high content microscopes (Fig 1). Our method enables fast imaging of up to 1,800 fish per day. Though we demonstrate DA neuron imaging of larval zebrafish in this work, the multi-pose imaging method can be generalized to the imaging of any cell type in the larval zebrafish brain.

## Results

### Posing of zebrafish larvae

**Fig 2:**
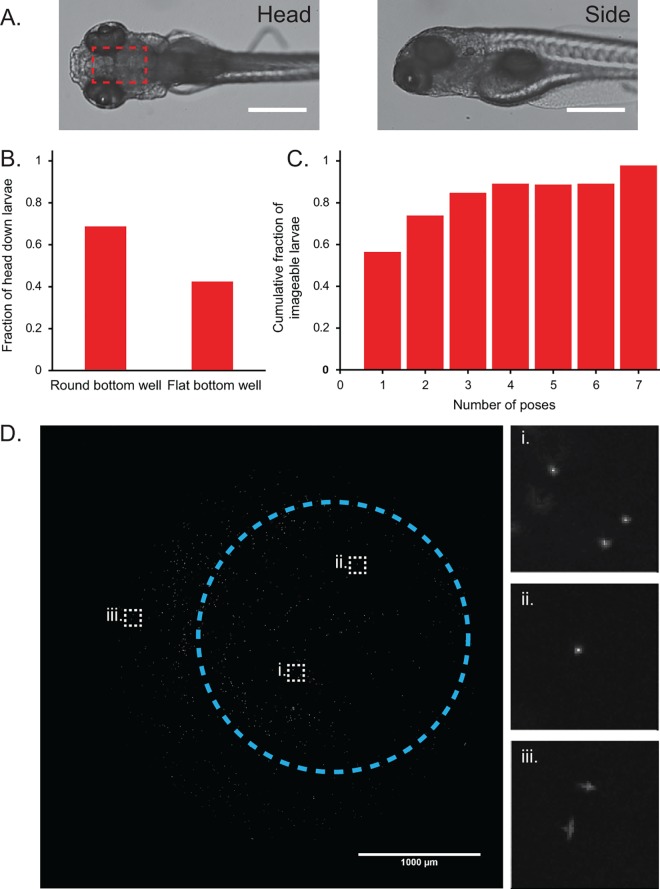
Round bottom 96-well plates preferentially orient larvae dorsal side down. (A) Zebrafish larvae must be oriented dorsal side down to image the brain region (boxed in red) using an inverted microscope. Larvae oriented side-down have their brains obscured by their large, pigmented eyes (scale bar = 500um). (B) Plots showing that round bottom 96-well plates preferentially orient fish dorsal side down for brain imaging (n = 80 larvae). (C) Approximately 90% of the larvae have at least one dorsal side-down image by the fourth reposing cycle (n = 46 larvae). (D) Maximum z-projection of 0.5um fluorescent beads used to characterize the aberrations caused by the round bottom well on an IN Cell 2000 system using a 4× 0.2 NA objective (scale bar: 1000um). Circled region indicates the typical area that a larval head rests, with the center of the circle marking the approximate center of the well. Insets show zoomed-in images of beads at different regions of the well. (i) and (ii) show no noticeable aberrations, whereas astigmatism and coma aberrations near the edges of the well are visible in (iii).

A critical step in imaging a zebrafish larva is properly positioning it such that the structure of interest is in clear view. Specifically, to image the larval brain, the larva needs to be posed such that its dorsal side faces the objective of an inverted microscope and its pigmented eyes do not obscure the brain region of interest (Fig 2A). We have observed that anesthetized embryos tend to sink headfirst, but only 42.5% of the larvae had the correct dorsal-side-down orientation after sinking to the bottom of the wells in flat bottom 96-well plates (Fig 2B). We looked at other plate designs, specifically round bottom plates, to see if the posing rate could be improved. In the case of the round bottom plates, 68.8% of the larvae were oriented correctly (p = 8.3 × 10^−4^, Fig 2B). We also found that round bottom plates had the added advantage of guiding the head into the center of the well, removing the need to search for the larvae within the well during imaging.

The correct-posing rate was still too low, however, for high-throughput applications. We discovered that using a liquid handler to quickly aspirate and replace 40uL of liquid in each well caused the anesthetized embryo within to tumble and assume a new pose. Therefore, we were able to increase the chance of acquiring an image of a correctly posed larva by repeatedly re-posing and imaging the larvae. In our test, 89% of the larvae had at least one good pose by the fourth posing cycle (Fig 2C). Considering practical robustness, we took five poses per larvae in our subsequent experiments.

We further examined whether the curved bottom of the well introduces optical aberrations that impair the imaging quality. We compared the full-width at half-maximum (FWHM) of point spread functions in round and flat bottom plates by imaging 0.5 μm fluorescent beads using our screening microscope (IN Cell 2000 high-content microscope with a 4× 0.2 NA objective). Within a ~ 2.2 mm diameter circle around the center of the well, where the larval head typically rests, we observed only a very minor difference in the average FWHM (1.53 μm for round bottom wells and 1.41 μm for flat ones) (Fig 2D). This result indicates that the geometry of the wells is well-suited for our applications, and that our image resolution is sufficient to resolve individual cells in the larval brain.

### Zebrafish larva screening

**Fig 3:**
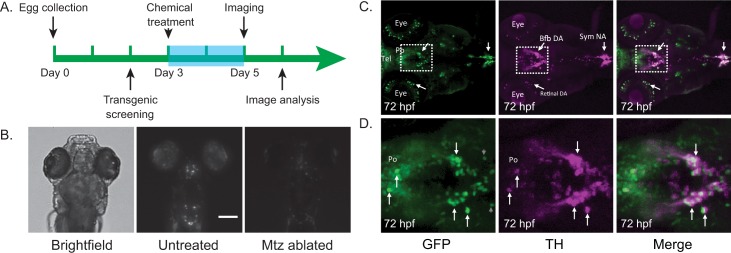
Chemo-genetic model screening details. (A) Timeline showing the details of fish husbandry and chemical treatment. Larvae were treated with metronidazole at 3dpf and imaged *in situ* at 5dpf. (B) The nitroreductase-metronidazole method is able to specifically ablate dopaminergic neurons, which express mCherry, in 5dpf larvae (scale bar = 200um). Images are of zebrafish larvae in the dorsal-down position. (C) Anti-tyrosine hydroxylase and anti-GFP antibody staining of 3dpf zebrafish embryos show good overlap in the ventral forebrain region DA neurons. bfb DA, basal forebrain dopaminergic neurons; Po, preoptic region; sym NA, sympathetic noradrenergic neurons; Tel, telencephalon; retinal DA, retinal dopaminergic neurons. (D) Zoomed-in views of areas boxed in (C).

We performed the multi-pose imaging method using an IN Cell 2000 high-content microscope and applied it to our DA neuron degeneration model. The IN Cell system is an automated microscope designed for high-throughput screening applications; for the purposes of our method, the only required functions are the ability to automatically move between wells in a 96-well plate and acquire fluorescence and bright field images at multiple z-depths. We screened and collected 2dpf (days post-fertilization) homozygous embryos, distributed them into 96-well plates with one fish per well, treated with Mtz or DMSO at 3dpf, and performed imaging at 5dpf (Fig 3A). With one bright-field image and a fluorescent image stack acquired for each fish (Fig 3B), it took 15 min to image through one 96-well plate, while the re-posing operation between acquisitions took approximately 1 min. Overall, five cycles of imaging and re-posing took a total of 75 minutes per plate, allowing > 1,800 fish larvae to be analyzed per day. In this case, our screening throughput was primarily limited by the production rate of transgenic fish embryos and not by image acquisition speed.

### Automated detection and analysis of brain region

**Fig 4:**
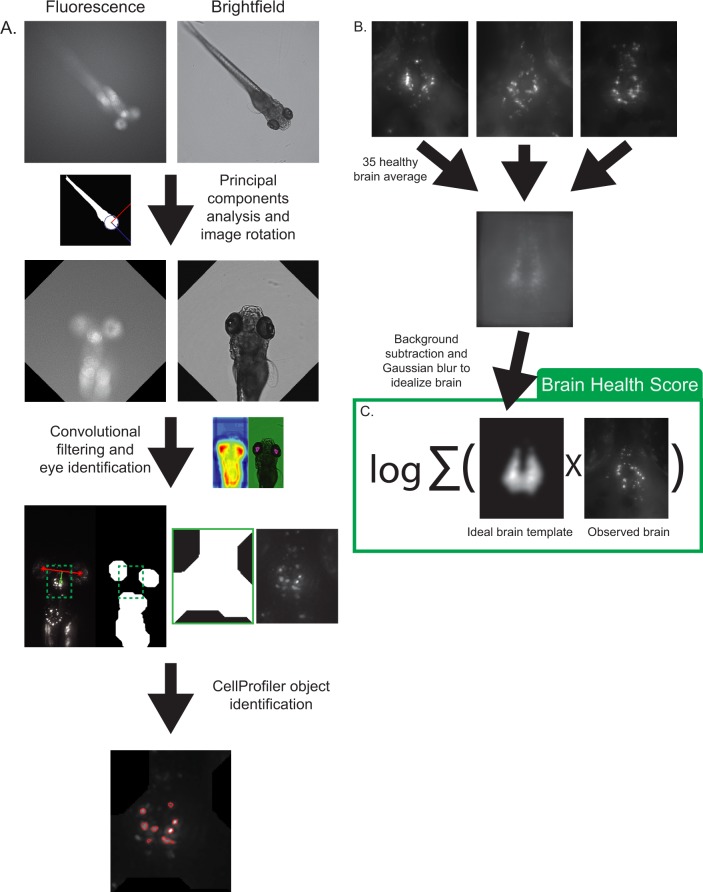
Image processing pipeline. (A) The fluorescence image is thresholded and a principal component analysis is used to rotate both images to a head-up position. The brightfield image is then thresholded and convolved with an eye-like filter to locate the eyes. The brain region is then identified using the eyes as landmarks, the image is cropped, and a maximum intensity z-projection is exported to CellProfiler for neuron identification. Red asterisks denote centroids of identified eye regions and the green box represents the final cropped area sent to CellProfiler for analysis. (B) Creation of an idealized brain template image. 35 healthy brains were registered and averaged in ImageJ. Background was subtracted and a Gaussian blur filter was applied to smooth and idealize the image. (C) The brain health score was defined as the logarithm of the covariance between an idealized brain template and an observed brain image.

With each larva imaged in five unique poses, we manually selected the best pose for subsequent automated analysis, taking into account the position of the eyes and visibility of the brain. We have developed a MATLAB script that identifies the in-plane image of the larva, rotates the image such that the larva adopts a head-up orientation, identifies the head, finds the eyes in the image, and then locates the brain using the eyes as landmarks (Fig 4). Correct identification of the brain is confirmed by a human and misidentifications, if any, are manually corrected. The maximum intensity z-projection of the fluorescence image of the brain for each larva was then exported to CellProfiler, a free open-source program designed to analyze images produced in high-throughput screens [21], for segmentation and quantification of DA neurons. A “brain health score” (BHS) was then calculated for each image, defined as the logarithm of the covariance between the image and an image of an idealized healthy brain.

### Evaluation of screening performance

**Fig 5:**
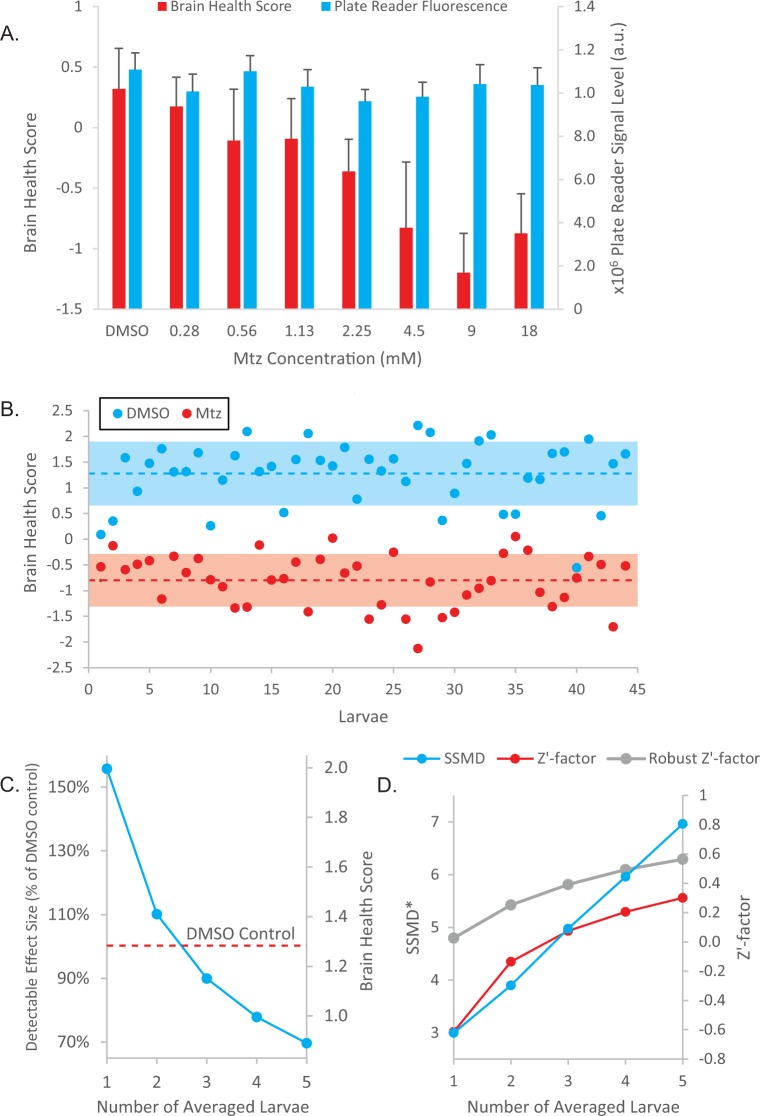
Screening metrics for the multi-pose imaging method. (A) Dose response of Mtz treatment. 7 dpf larvae treated with a serial dilution of Mtz were imaged using each technique and BHS values were plotted. Error bars represent 95% confidence intervals. (B) BHS returned using the multi-pose imaging method on 5 dpf untreated larvae and larvae treated with 9 mM Mtz. Shaded areas mark standard deviations. The SSMD* score of our assay is 2.58. (C) The number of fish averaged per condition sets the minimum detectable effect size relative to the DMSO controls, assuming a false positive rate of 0.5% and false negative rate of 10%. (D) Estimated screening metric scores as a result of averaging data from multiple larvae on bootstrapped data (1000 iterations). SSMD*, Z’-factor and robust Z’-factor all dramatically increase as more larvae are averaged together.

We imaged larvae treated with a serial dilution of Mtz using our multi-pose imaging method and observed a clear dose response of Mtz-dependent DA neuron ablation (p = 1.95 × 10^−9^ using ANOVA) (Fig 5A). In contrast, when the same set of samples were analyzed by a plate reader for total fluorescence intensity, Mtz-treated and untreated larvae cannot be differentiated at any concentration of Mtz (p = 0.09 using ANOVA). The ineffectiveness of the plate reader can likely be attributed to the small size of the region of interest (the brain region) and the comparatively high autofluorescence of the larvae (e.g. the yolk sac), which demonstrates the necessity of our targeted, high-resolution imaging method to detect this phenotype change.

Next, we evaluated the effectiveness of our chemo-genetic DA neuron ablation assay by splitting the larvae into Mtz treated and untreated groups and quantified neuronal health using the multi-pose imaging method. Z’-factor, a dimensionless number that measures the separation between the positive and negative controls, was initially used to quantify the quality of the assay. The Z’-factor of our assay using individual fish was -0.64, within the range of other similar zebrafish screens (from -7.1 to 0.78) [12]. Considering that Z’-factor assumes the data is normally distributed and is susceptible to outliers, we also calculated alternative measures of screen quality, which include the robust Z’-factor and the robust strictly standardized mean difference (SSMD*) score. These metrics are more suited as quality control measures for whole-organism screens that often have large data outliers [22–25]. Our screen has a robust Z’-factor of 0.07 and a SSMD* score of 2.58, which corresponds to a good assay with a strong control [23,26].

Performing a drug discovery screen requires tight control over the false positive and negative rates. To screen libraries of up to 10^5^ compounds, it is desirable to have no higher than 0.5% false positive and 10% false negative rates, which would result in an expected 500 false positives for rescreening. With this standard, averaging the score from at least 3 larvae per condition is necessary to identify hits that fully restore BHS to the DMSO control level, whereas averaging 5 larvae allows reliable detection of hits that restore the brain health score to 70% between the DMSO control and full Mtz treatment. Correspondingly, SSMD* is increased to 6.96 (robust Z’-factor = 0.56) (Fig 5D).

## Discussion

Drug discovery in whole-organisms, such as zebrafish, offers a number of advantages over *in vitro* or cell culture-based approaches; for example, toxicity and polypharmacology, difficult to assay *in vitro*, are directly assayed at the whole-organism level. However, whole organism screens are still relatively rare, due in large part to lower throughput, higher cost, and relative novelty compared to traditional approaches. Our multi-pose imaging method addresses many of these challenges. Our technique is robust and has a throughput of approximately 1,800 fish per day. We have validated our model in a chemogenetic model of DA neuron degeneration, showing that it is sensitive enough for screens in library sizes up to 10^5^ compounds (a scale at which embryo production becomes the rate-limiting step). Most importantly, the multi-pose imaging method is simple to implement, requiring only an automated microscope and a liquid handler, standard equipment already present in most screening facilities.

There remain a number of possible improvements to the multi-pose imaging method. Acquiring images of the larval brain represents an extremely rich data source. Features such as the shape, size, and patterning of the neurons in space are potentially informative. We currently use the brain health score as a relatively simple measurement to identify hit compounds, but more sophisticated multi-parametric models that utilize multiple features can increase the sensitivity and specificity of our assay. Fish husbandry remains a large area of improvement for any zebrafish screen, and our assay was bottlenecked by the production of transgenic larvae. A large scale screen would require the maintenance of large numbers of transgenic adults and improved crossing techniques capable of generating the thousands of larvae a day required for screening.

## Methods

### Establishment of a Chemo-genetic dopaminergic neuron degeneration model in larval zebrafish

To gain genetic access of dopamine neurons in larval zebrafish, we used the 5’ and 3’ regulatory elements from the Fugu tyrosine hydroxylase (*th)* locus, reasoning that the compact Fugu genome likely leads to more closely juxtaposed regulated elements than that in zebrafish. Seven kilobases of sequences flanking the Fugu *th* gene were amplified from a Fugu BAC and cloned into a Tol2 vector flanking gal4uas-EGFP sequences. The plasmid was micro-injected into 1-cell stage zebrafish embryos, which were raised to adulthood. Germ-line transgenic founders were identified through genetic crossing and visual screening for fluorescent DA neurons under a Leica fluorescent stereomicroscope. The *Tg[fuguth-gal4uas-EGFP]* transgenic line (allele number: s2509) was then established and verified by double immunostaining with anti-TH and anti-GFP antibodies, which recapitulated the endogenous *th* expression in the ventral forebrain region (Fig 3C and 3D). A double transgenic line was created by mating *Tg[fuguth-gal4uas-EGFP]* with *Tg[uas-NTRmcherry]* [27]. The tg[uas-NTRmcherry] line was obtained from ZIRC. Larvae were exposed to 9 mM MtzZ for 24–48 hours, resulting in specific ablation of DA neurons.

### Fish husbandry

All adult zebrafish were raised at the UCSF zebrafish facility at 28°C under a 14/10 hour light/dark cycle. Embryos used in experiments were created by pairing one transgenic and one wild-type fish in a breeding tank separated by a divider. The fish were left overnight and the divider removed at 9AM the next morning. The eggs were collected at 10 AM and cultured in blue egg water with 75 μM 1-phenyl 2-thiourea (PTU) for 48 hours to inhibit pigment production and facilitate imaging. At 2 days post-fertilization (dpf) the embryos were screened for fluorescence under a dissection microscope to confirm DA expression of the NTR:mCherry transgene before being used in further experiments. All Mtz treatment experiments were performed by incubating larvae in 9 mM Mtz in 0.2% DMSO and blue egg water for 48 hours. All work followed the NIH guidelines and was approved by the University of California San Francisco Committee on Animal Research.

### Posing comparison between plate designs

200uL of 16mg/100 mL tricaine methanesulfonate in blue egg water was added to a 96-well round bottom plate (Corning) and one 5dpf larvae pipetted into each well (n = 80 larvae). The fish were inspected by eye under a dissection microscope and visually scored as either head down, side down, or floating (all data on wells with floating fish were discarded). The same larvae were then transferred to a standard 96-well plate (MatriPlate 96-well glass bottom) filled with the tricaine solution and with each larvae occupying its corresponding well. The larvae were then visually scored again. The proportion of larvae in the correct dorsal side down and incorrect lateral side down categories in each plate condition were then compared using a Chi-squared test in Excel.

### Minimal pose experiment

5dpf larvae were added to a 96-well round bottom plate and anesthetized in 16mg/100mL tricaine solution (n = 46 larvae). The larvae were imaged in bright-field and reposed seven times using the parameters described in the image acquisition section. The bright-field images were scored as either head down, side down, or not imageable (usually out of focus or missing from the field of view). The pose of the first head down image for each larvae was recorded, and the cumulative fraction of larvae that had at least one good pose was plotted against the cumulative number of poses (Fig 2C).

### Image acquisition

Images were acquired using an IN Cell 2000 automated microscope (GE Healthcare). Seven fluorescent z-sections spaced 100 microns apart (500ms exposure) was acquired of each well in the TexasRed channel using a 4x 0.2NA objective (Nikon) using the built-in 2.5D deconvolution setting. A brightfield image was also acquired of the well at zero offset (10ms exposure). Upon completion of imaging, plates were moved to a liquid handler (Biomek FXp) that mixed 40uL of the solution in each well to reorient the larvae within. A 96-channel head using P20 pipette tips was used in the liquid handling operation. The plates were then returned to the microscope for another round of imaging. Each plate underwent five cycles of imaging and reorientation to maximize the probability of acquiring an image of the larva in the correct pose.

The resolution of the system was determined by acquiring images of 0.5um red fluorescent beads (FluoSpheres F8887, Molecular Probes) embedded in 1% agarose in round bottom 96-well plates, as well as flat bottom 96-well plates (Falcon). A 50um thick z-stack with 1um steps was acquired using the above image acquisition parameters and a maximum intensity z-projection was performed for both plate types. We then measured the 2D PSF of in-focus beads within the posing area (circled area in Fig 2D) in ImageJ using the “PSF Tool 2D” within the MosiacSuite plugin. The FWHMs for each plate were then compared in Excel using a Welch’s t-test (n_round_ = 132 PSFs, n_flat_ = 118 PSFs).

### Image analysis

Images were analyzed in two phases, the pre-processing phase and the feature extraction phase. In the pre-processing phase, the images were loaded into a custom Matlab script that presents the five poses associated with a given larva to the user. The user manually selects the best pose, accounting for the position of the eyes and view of the brain region (only poses that are dorsal side down with a clear view of the brain region were selected for analysis). Next, an automated image processing algorithm analyzes the fluorescence and brightfield images to extract a clear image of the brain region. First, a maximum intensity projection is made of the fluorescence image stack. Second, the brightfield image is resized to half its original size to speed up identification of gross anatomical features, such as the head and eyes. Third, the head region of the fish is identified in the first (usually out of focus) image in the fluorescence z-stack by using a circular Hough transform looking for circular features between 80 to 150 pixels in diameter. The fluorescence image is thresholded using Otsu’s method, and a principal components analysis is run on the binarized image to identify the long axis of the fish (cranial-caudal axis). Both fluorescence and brightfield images are then rotated such that the long axis of the fish is at zero degrees. Fourth, a black oval 60 x 90 pixels is convolved with the brightfield image and the result thresholded using Otsu’s method. The centroids of the two largest regions are marked as the eyes. Fifth, a line is drawn between the two eyes, and a region 35 pixels from the midpoint of the line in the perpendicular direction is marked as the center of the brain. Lastly, the coordinates of the brain center are transformed into the coordinates of the original, full size fluorescence image and a region 190 x 220 pixels centered on the brain is cropped. The brightfield image is also thresholded using Otsu’s method and cropped in the same manner to create an image mask that identifies the eye regions. The image of the brain and the mask of the eyes are then saved for further analysis. Once all fish have been processed, the results are displayed to the user to either accept or reject the automated analysis. Any fish that were not successfully processed are then manually rotated and cropped.

In the feature extraction phase the pre-processed images are first loaded into CellProfiler. The eye mask created using the brightfield image is overlaid on top of the fluorescence image to remove the autofluorescence due to the eyes from the image. The fluorescence image is then smoothed using the “Smooth” function with a Gaussian filter that has a full width at half-maximum of 12 pixels. This image is then subtracted from the original image using “ImageMath” in order to remove the background in the image while preserving the neuronal signal. The neurons are then identified using “IdentifyPrimaryObjects” searching for objects between 5 and 20 pixels in diameter and using a two class Otsu threshold with a threshold correction factor of 5. These identified neurons are then overlaid on top of the original fluorescence image and various features such as the area, total fluorescence, mean fluorescence, etc. are extracted and saved to a spreadsheet for hit identification.

### Determination of brain health score

A list of features returned by CellProfiler were analyzed to see which feature best distinguished the treated and untreated larvae. Welch’s t-tests were run on each feature and the brain health score was chosen for its high statistical significance (p = 10^−29^) and biological significance (the score quantifies both intensity and shape of the brain). The brain health score (BHS) was defined as the logarithm of the covariance between the brain image and a template image: *BHS* = *log*_2_ ∑_*i*,*j*_ *I_ij_M_ij_*, where *I* is the pixel intensity of the image and *M* is the pixel intensity of a template image at pixel *i*, *j* (Fig 4C). The template image was generated by averaging 35 images of healthy brains. BHS is a measure of how much a given image resembles a healthy, idealized brain in both intensity and shape; healthy brains will have large pixel intensities where pixels values in the template image are high resulting in a large summation value and vice versa. Calculation of the brain health score was done in CellProfiler using the “ImageMath” and “MeasureImageIntensity” modules.

The idealized brain template was created by taking 35 maximum intensity z-projections of untreated 5dpf larvae, further registering the images using the “Rigid Body Transform” in the ImageJ StackReg plugin, finding the average z-projection of the registered image stack, using the Subtract Background ImageJ command (rolling ball radius of 150 pixels), and finally filtering the image using a Gaussian blur with a sigma of 7 pixels in order to smooth the image and further idealize it. The image was then scaled to values between 0 and 1 and saved (Fig 4B).

### Comparison of multi-pose imaging and plate-reader methods

6dpf larvae were loaded into three 96-well plates with one larvae per well. Each row of the plate was dosed with a two-fold serial dilution of Mtz ranging from 18 mM at the highest concentration, to 281 μM at the lowest, with the last row receiving no Mtz. All wells had a final DMSO concentration of 0.2%. The larvae were incubated at 28°C for 24 hours before being anesthetized by 16 mg / 100 mL tricaine. Floating larvae were removed from the assay–a total of 252 larvae were used in this assay with an average of 32 larvae per condition. The larvae were first imaged on an Envision 2104 plate reader (Perkin Elmer) with a 1 mm measurement height, 100 flashes, and a PMT gain of 150. The plates imaged using the multi-pose method as described in the image acquisition and analysis sections. The plate-reader measurements and the multi-pose method data were each analyzed using one-way ANOVA tests in Microsoft Excel to determine if any statistically significant differences were detected between the various Mtz concentrations using each method.

## Z’-factor, SSMD*, and sample size calculations

3dpf larvae were loaded into a 96-well plate with one larva per well. Half of the plate (rows A-D) were treated with 0.2% DMSO in blue egg water and the other half (rows E-H) were treated with 9 mM Mtz in 0.2% DMSO and blue egg water. 44 larvae were used for each condition (4 larvae in each condition died of unknown causes) for a total of 88 larvae in one plate. After loading, the larvae were then incubated at 28°C for 48 hours. At 5dpf, the larvae were anesthetized by adding tricaine to each well to reach a final concentration of 16mg/100mL. The anesthetized larvae were imaged using an IN Cell 2000 microscope and analyzed using the MATLAB and CellProfiler scripts described in the image acquisition and analysis sections.

The brain health score was compared between Mtz treated and untreated larvae and the Z’-factor was calculated using the formula: $Z^{\prime }=1-\frac{3*(\sigma_{+}+\sigma_{-})}{\left|\mu_{+}-\mu_{-}\right|}$. μ and σ refer to the mean and standard deviation respectively and + and–to embryos treated with DMSO-only and 9 mM Mtz respectively. The robust Z’-factor was calculated using the same formula, but using the sample medians and median absolute deviations instead of mean and standard deviations respectively. SSMD* was calculated using the formula ${SSMD}^{*}=\frac{{\tilde{X}}_{+}-{\tilde{X}}_{-}}{1.4826\sqrt{{{\tilde{s}}_{+}}^{2}+{{\tilde{s}}_{-}}^{2}}}$ where $\tilde{X}$ and $\tilde{s}$ represent the median and median absolute deviation of the DMSO and Mtz controls respectively [26].

The relationship between larval sample size and the detectable effect size (Fig 5C) was calculated using the formula: $n=\frac{\left[2\times {\left(Z_{\alpha }+Z_{1-\beta }\right)}^{2}\times \sigma^{2}\right]}{\Delta^{2}}$ where n is the sample size, Z_α_ = 2.81 and Z_1-β_ = 1.28 (corresponding to a 0.5% false positive and 10% false negative rate respectively), *σ* is the pooled standard deviation of the DMSO and Mtz controls, and Δ = *BHS*(measurement)–*BHS*(Mtz) is the effect size [28]. The normalized effect size (Fig 5C) is calculated by dividing Δ with *BHS*(DMSO)–*BHS*(Mtz).

The effects of averaging upon Z’-factor and SSMD* were simulated by bootstrapping the data into 1000 bootstrapped samples. The observations within each bootstrapped sample were randomly assigned into groups ranging from 1 to 5 samples each. Each group of observations was then averaged, and the group averages used to calculate the Z’-factor and SSMD* score for its corresponding bootstrapped sample. A final Z’-factor and SSMD* score were determined finding the average Z’-factor and SSMD* across bootstrapped samples and reported in Fig 5D.
